# Accumulation Patterns and Health Risk Assessment of Trace Elements in Intermuscular Bone-Free Crucian Carp

**DOI:** 10.3390/toxics13070595

**Published:** 2025-07-16

**Authors:** Shizhan Tang, Na Li, Zhipeng Sun, Ting Yan, Tingting Zhang, Huan Xu, Zhongxiang Chen, Dongli Qin, Youyi Kuang

**Affiliations:** 1 Heilongjiang River Fisheries Research Institute, Chinese Academy of Fishery Sciences, Harbin 150070, China; tangshizhan@hrfri.ac.cn (S.T.); lina01251@163.com (N.L.); sunzhipeng@hrfri.ac.cn (Z.S.); yanting@hrfri.ac.cn (T.Y.); zhangtingting@hrfri.ac.cn (T.Z.); xuhuan@hrfri.ac.cn (H.X.); chenzx@hrfri.ac.cn (Z.C.); qindongli@hrfri.ac.cn (D.Q.); 2 Supervision, Inspection and Testing Center for Fishery Environment and Aquatic Products (Harbin), Ministry of Agriculture and Rural Affairs, Harbin 150070, China

**Keywords:** trace elements, gene-edited fish, health risk assessment, pollution index, crucian carp

## Abstract

This study investigated the accumulation characteristics and associated health risks of 11 trace elements (Al, Rb, Cr, Ni, Mo, Sr, Pb, Ba, Ag, As, and Ga) in four crucian carp varieties: gene-edited intermuscular bone-free crucian carp (*Carassius auratus,* WUCI) and its sibling wild-type (*Carassius auratus*, WT), Fangzheng silver crucian carp (*Carassius gibelio var* Fangzheng, FZYJ), and Songpu silver crucian carp (*Carassius gibelio var* Songpu, SPYJ). Results showed that Al and Rb were the most abundant elements across all groups. WUCI exhibited distinct accumulation patterns, including significantly higher hepatic Mo concentrations (0.265 ± 0.032 mg/kg) and muscle/liver Rb levels (muscle: 8.74 ± 1.21 mg/kg; liver: 12.56 ± 2.05 mg/kg) compared to other varieties (*p* < 0.05), which supports the hypothesis of genotype-specific differences in heavy metal accumulation. Correlation analysis revealed that WUCI exhibited similar elemental interactions with WT and SPYJ (e.g., Al-Ni positive correlation, |rs| ≥ 0.8), while SPYJ displayed distinct patterns with fifteen negative correlations compared to three to five in others varieties, suggesting a potential alteration in elemental homeostasis. Pollution index (*P_i_*) assessments indicated mild contamination for Pb in SPYJ liver (*P_i_* = 0.265) and Cr/As in WUCI muscle (*P_i_* = 0.247/0.218). Despite these values, all hazard indices remained below the established safety thresholds (*THQ* < 0.1, *HI* < 0.25, *TCR* < 10^−6^), reinforcing the overall safety of the tested fish. Notably, muscle As levels (0.86 ± 0.15 mg/kg) exceeded hepatic concentrations (0.52 ± 0.09 mg/kg), potentially due to differential detoxification mechanisms. These findings demonstrate the food safety of all tested varieties, while highlighting genotype-specific metabolic adaptations, providing critical data for evaluating gene edited aquatic products.

## 1. Introduction

The crucian carp (*Carassius auratus*), from the family Cyprinidae, is an economically important valuable freshwater species, prized for its adaptability, rapid growth, high reproductive capacity, and nutritional value [1,2,3]. However, numerous intermuscular bones (IBs) present significant challenges: increasing choking hazards, complicating food processing, and potentially reducing meat freshness, all of which diminish its commercial value [4]. To address these issues, selective breeding, hybridization, gynogenesis etc. was applied in cyprinids including the crucian carp to create new strain with fewer IBs [5,6]. While these advances represent progress, complete IB elimination remained elusive until the application of gene-editing technologies targeting key osteogenic genes (*runx2b* and *bmp6*), which successfully produced a novel gene-edited intermuscular bone-free crucian carp strain (WUCI) [7,8,9]. This breakthrough not only improves culinary quality but also provides valuable insights for quality improvement in other cyprinid species.

Although WUCI demonstrates clear advantages in processing efficiency, the food safety was not comprehensive evaluated. Our prior studies identified significant bioaccumulation changes in seven essential metals (Mg, Mn, Fe, Cu, Zn, Se, K) in WUCI crucian carp [8]. Specifically, hepatic Fe and Se levels were elevated compared to wild-type (*Carassius auratus*, WT) controls, while muscular profiles remained unchanged [8], implying genotype-dependent metabolic modulation. These findings underscore the critical necessity for systematic multielement analysis to comprehensively evaluate potential food safety implications.

The biological roles of metallic elements in fish physiology demonstrate a complex duality: while essential trace elements like copper (Cu) and zinc (Zn) play crucial roles in enzymatic functions, hematopoiesis, and immune response [10,11,12,13], toxic heavy metals such as cadmium (Cd) and lead (Pb), when accumulated, tend to impair antioxidant defenses and can cause mortality at high concentrations in vital organs (e.g., gill and liver) [14,15]. Acute toxicity studies establish the following potency ranking: Cu > Cd > Zn > Cr [15]. Our previous studies have revealed that the accumulation patterns of elements such as Rb, Cr, Sr, Ba, Ag, and As are significantly influenced by geographical environmental differences, while Ga and Ni are primarily affected by electronic waste contamination in the environment. Additionally, Al accumulation may contribute to the development of neurodegenerative diseases, and Mo, as an essential trace element, plays a crucial role in enzymatic synthesis [16,17,18]. However, there remains a lack of data on the bioaccumulation of these elements in genetically modified aquatic species. Of particular concern is the potential for trophic transfer of these elements through human consumption, which necessitates rigorous monitoring of metal levels in food fish [17,18]. In recent years, the application of gene-editing technologies (e.g., bmp6 knockout in intermuscular bone-less crucian carp) in fish breeding has raised concerns about potential alterations in heavy metal metabolism, which may involve dysregulation of ion transport systems and impairment of detoxification mechanisms [8]. However, current research on heavy metal accumulation in gene-edited fish remains limited. Notably, safety assessments of commercially available gene-edited fish varieties (such as genetically modified salmon in the United States) have primarily focused on growth performance traits, while systematic analyses of elemental accumulation patterns are conspicuously lacking [6].

Building upon our previous findings on the concentrations of seven essential elements in WUCI liver tissue, this study extends the investigation by systematically comparing the trace element profiles in both muscle and liver tissues across four genetically distinct fish strains, including WT, WUCI, Fangzheng silver crucian carp (*Carassius gibelio var* Fangzheng, FZYJ), and Songpu silver crucian carp (*Carassius gibelio var* Songpu, SPYJ). We further assess potential contamination levels and conduct comprehensive health risk evaluations to thoroughly address the implications for food safety. These investigations not only fill critical knowledge gaps regarding elemental metabolism in genetically modified carp, but also provide valuable methodological frameworks for future research on IB free cyprinids.

## 2. Materials and Methods

### 2.1. Experimental Materials

The WUCI strain used in this study was generated and maintained in our laboratory through a targeted knockout of the Bmp6 [8]. The reproduction and larval culture of WUCI strain was similar to our previous study [8] and simply described as below. Parental fish were reared in a 700 m^2^ outdoor pond. These fish were injected with specific oxytocin drugs to induce spawning, including luteinizing hormone-releasing hormone (LRH-2, 4 μg/kg body weight); domperidone (DOM, 1 mg/kg body weight), and human chorionic gonadotropin (HCG, 100 U/kg body weight). The fertilized eggs were then incubated in 24 cm × 20 cm × 24 cm incubation tanks at a control condition with a constant temperature range of 22–23 °C. After hatching, larvae were fed Artemia 4 times/day until they reached a body length of 2–3 cm (SL). Upon reaching this size, 1000 larvae were then transferred to a 500 m^2^ outdoor pond and fed artificial feed twice a day. The wild-type (WT), Fangzheng silver crucian carp (FZYJ), and Songpu silver crucian carp (SPYJ) strains were all kept in our laboratory, the reproduction and larval culture was conducted at the same time with the same procedure to the WUCI strain. All four crucian carp strains were cultured at the Hulan Experimental Station of Heilongjiang Fisheries Research Institute of Chinese Academy of Fishery Sciences (HRFRI).

### 2.2. Sample Pretreatment and Analysis

The experimental trial was conducted over a 4-month culture period (April–August 2023). At harvest, ten representative fish per strain (WUCI, WT, FZYJ, and SPYJ) were randomly sampled for analysis. Biometric measurements of sampled specimens are summarized in Table 1. Their muscle and liver tissues were homogenized. The samples were stored at −20 °C before trace element analysis. The tissue sample preparation protocol was developed in our research laboratory and validated for crucian carp analysis [17,18]. Briefly, 0.5 g of muscle or liver tissue was digested with 5 mL HNO_3_ (65%, trace metal grade) and 2 mL H_2_O_2_ (30%, ultrapure) using a microwave digestion system (MARS X, CEM, Matthews, NC, USA). The digestion program consisted of: (1) ramp to 120 °C (10 min hold); (2) ramp to 180 °C (15 min hold); and (3) cooling to 50 °C. After digestion, the acid was evaporated to 0.5 mL, and the volume was adjusted to 10 mL with ultrapure water. Samples that exceeded the linear range of the standard curve were diluted and reanalyzed. Subsequently, the solvent and reagent blanks were analyzed using an Inductively Coupled Plasma-Mass Spectrometer (ICP-MS) 7500cx (Agilent, Santa Clara, CA, USA), equipped with an eight-stage rod collision/reaction cell system. Eleven elements (Al, Rb, Cr, Ni, Mo, Sr, Pb, Ba, Ag, As, Ga, Cd, Cu) were selected to reveal the presence of trace elements in crucian carp in the present study. The detailed detection procedures and quality control were as described in our previous study (Table S1) [17,18]. For quality assurance, 5% to 10% of the samples were randomly selected and analyzed six times to ensure repeatability. A certified reference material (prawn, GBW 10050), provided by the Chinese Institute of Geophysical and Geochemical Survey, was used to verify the determination of this standard, and recoveries between 90.0% and 110% were obtained (Table S2). The relative standard deviations (RSDs) and measurement uncertainties for all elements were lower than 10% (Table S2 and Table S3). The final data were calculated based on wet weight.

### 2.3. Heavy Metal Pollution Evaluation

For pollution assessment, five regulated heavy metals (Cu, Pb, Cd, Cr, and As) were selected based on their prevalence in aquatic environments and their potential risk to human health, in accordance with China’s food safety standards. These metals were evaluated using the single-factor pollution index (*P_i_*) method [18]:

(1) $$P_{i}=\frac{C_{i}}{C_{si}}$$

where *P_i_* is the pollution index for the *i*-th heavy metal in crucian carp tissues; *C_i_* is the measured concentration of the *i*-th heavy metal (mg/kg wet weight); and *C_si_* refers to the maximum permissible limit for the *i*-th heavy metal according to Chinese regulatory standards (mg/kg).

Reference thresholds are based on NY 5073-2006 (Maximum levels of toxic and hazardous substances in pollution-free aquatic products), GB 2762-2022 (National food safety standards—Maximum levels of contaminants in foods).

Pollution levels were classified based on the established pollution index criteria for aquatic products as follows:

*P_i_* < 0.2: Background level (unpolluted)

0.2 ≤ *P_i_* < 0.6: Mild pollution

0.6 ≤ *P_i_* < 1.0: Moderate pollution

*P_i_* ≥ 1.0: Severe pollution

### 2.4. Health Risk Assessment

Methodology for assessing health risks of heavy metals using estimated daily intake (EDI):

(2) $$EDI=\frac{C_{i}\times D_{i}}{AW}$$

where *D_i_* denotes the daily intake rate of Carassius *auratus* for adult Chinese individuals; *C_i_* (μg/kg wet weight) represents the measured concentration of each heavy metal in fish tissue; *D_i_* (kg/day) is the daily consumption rate of crucian carp, estimated as 30.68 g/day based on China’s per capita aquatic product consumption [19]; and *AW* refers to the average body weight of Chinese adults (61.75 kg).

The target hazard quotient (*THQ*) was employed to assess potential non-carcinogenic health risks associated with heavy metal exposure through fish consumption (USEPA, 2000). The *THQ* is calculated as:

(3) $$THQ_{i}=\frac{EDI}{RfD}\times 10^{-3}$$

where *EDI* represents the estimated daily intake of metals (mg/day) and *RfD* denotes the oral reference dose (mg/kg/day), with established values for: Cu: 0.04, Zn: 0.05, Pb: 0.004, Cd: 0.001, Cr: 1.5 (USEPA, 2010). A *THQ* value ≥ 1 indicates potential health concerns [18]. The reference dose (*RfD*) represents the maximum daily exposure that is unlikely to cause adverse effects during a lifetime [18,19,20].

For the cumulative risk assessment of multiple metals, the hazard index (*HI*) was calculated as the sum of individual *THQ* values:

(4) $$HI=\sum_{i=1}^{n}THQ_{i}$$

Chronic exposure to carcinogenic metals was evaluated using the target cancer risk (*TCR*) model:

(5) $$TCR=\frac{EFr\times ED\times EDI\times CSF_{i}}{AW\times AT}\times 10^{-3}$$

where *EF_r_*: exposure frequency (365 days/year); *ED*: exposure duration (70 years for adults); *AT*: averaging time (70 years × 365 days); and *CSF*: cancer slope factor, with values: Cr: 0.5 mg/kg/day, Ni: 1.7 mg/kg/day, As: 1.5 mg/kg/day, Pb: 0.0085 mg/kg/day. According to USEPA guidelines [18,19,20], the recommended *TCR* range is 1 × 10^−6^ to 1 × 10^−4^, where:

*TCR* > 10^−4^ indicates unacceptable risk

10^−6^ < *TCR* < 10^−4^ suggests tolerable risk

*TCR* < 10^−6^ represents negligible risk

Here, the *TCR* values are used to assess the potential cancer risk associated with heavy metal exposure. It is crucial to note that a *TCR* > 10^−4^ would suggest a need for intervention or mitigation, as this level of exposure is considered hazardous to human health.

### 2.5. Statistical Analysis

The concentrations of trace elements (Al, Rb, Cr, Ni, Mo, Sr, Pb, Ba, Ag, As, Ga) in muscle and liver tissues of the four crucian carp strains were analyzed to assess their accumulation and enrichment patterns using R software (version 4.2.1). Inter-group differences were assessed by one-way analysis of variance (ANOVA), with post hoc multiple comparisons conducted using the least significant difference (LSD) method when significant differences were observed.

Data normality was verified by the Shapiro–Wilk test (*p* > 0.05), and homogeneity of variance was confirmed using the Levene’s test (*p* > 0.05). Spearman’s correlation analysis was employed to examine the relationships among trace element concentrations in different tissues across the four strains to identify potential accumulation patterns. A threshold of *p* < 0.05 was set for statistical significance.

## 3. Results and Discussion

### 3.1. Comparative Analysis of Trace Element Profiles in Four Crucian Carp Strains

**Figure 1:**
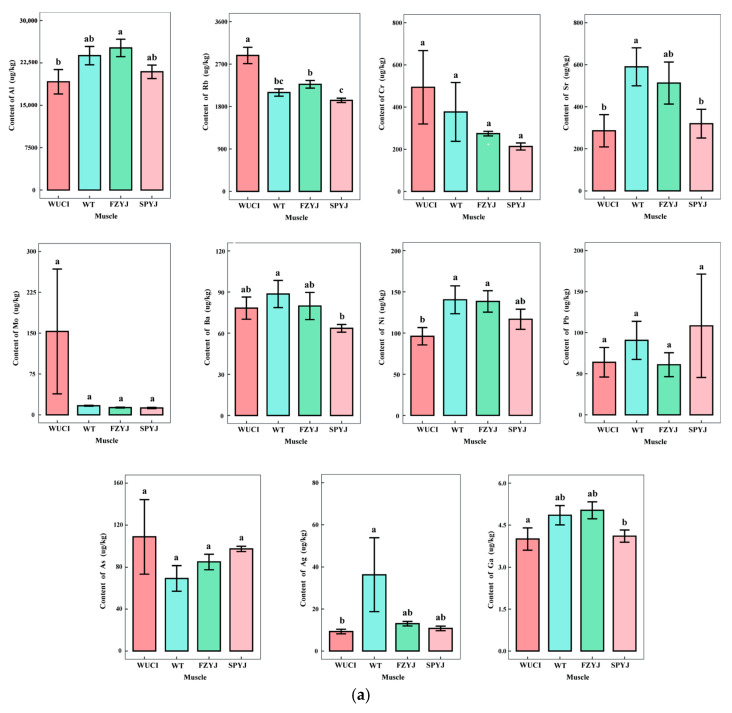

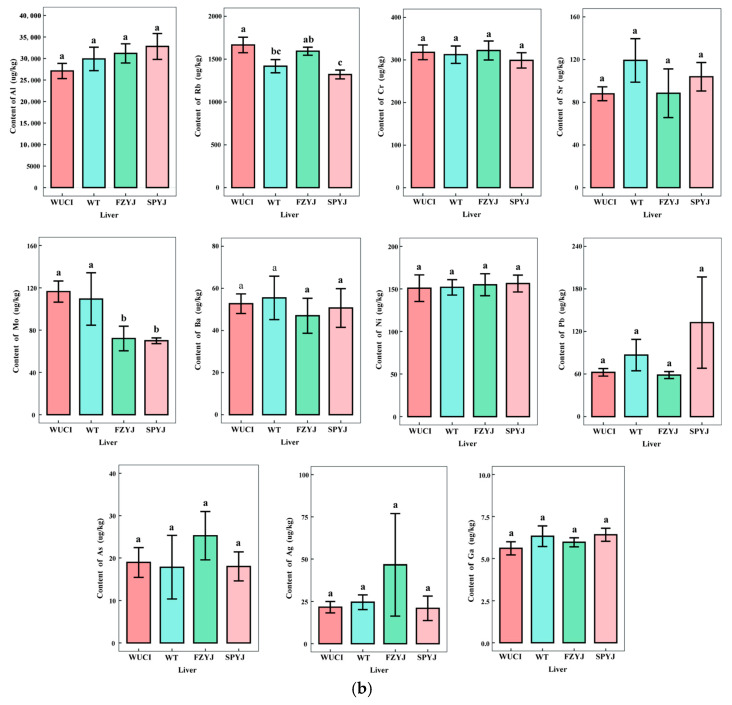
Concentrations of heavy metals (μg/kg wet weight) in tissues of four crucian carp strains. Shared letters indicate no statistically significant difference (*p* > 0.05) between groups; Different letters denote statistically significant differences (*p* < 0.05) between the corresponding groups. (**a**) Element concentration in muscle tissue; (**b**) Element concentration in liver tissue.

As an economically important freshwater fish, crucian carp serves as both a nutritional source of essential trace elements and a potential bioaccumulator of hazardous metals [21,22]. This study investigates the tissue-specific distribution of 11 elements (Al, Rb, Cr, Ni, Mo, Sr, Pb, Ba, Ag, As, and Ga) in muscle and liver tissues across four genetically distinct variants: gene-edited IB-free strain (WUCI) and its wild-type sibling (WT), Songpu silver crucian carp (SPYJ), and Fangzheng silver crucian carp (FZYJ). The analysis revealed Al and Rb as the predominant elements in both tissues, followed by Cr, Ni, Mo, Sr, and Pb, with Ba, Ag, As, and Ga occurring at trace levels (Figure 1a,b). Notably, WUCI exhibited unique accumulation patterns, including significantly elevated hepatic Mo (vs. FZYJ/SPYJ, *p* < 0.05) and Rb (vs. WT/SPYJ, *p* < 0.05), potentially linked to altered purine metabolism and ion transport regulation following bmp6 knockout [8,23]. Muscle tissue analysis showed WUCI exhibited reduced Al, Ni, Sr, Ag, and Ga, but increased Ba compared to other variants (*p* < 0.05). These patterns correlate with its slower growth phenotype and mirror findings in other aquatic species, where these elements associate with growth rate [6,18]. In this study, the concentrations of Ni, Cd, As, and Cr were consistent with previous reports. However, Pb levels in Spanish freshwater fish exceeded our measurements by more than 5-fold. This substantial discrepancy likely results from localized pollution sources, particularly the ingestion of spent Pb shot pellets—a contamination pathway well-documented in waterbirds [24]. These results demonstrate that both genetic modification (particularly growth-affecting edits such as bmp6) and inherent growth characteristics significantly influence elemental metabolism, underscoring the importance of conducting comprehensive safety assessments that evaluate both nutritional quality and potential toxicological risks in genetically engineered aquatic products. The distinct elemental profiles observed, especially WUCI’s higher Rb and Mo levels coupled with reduced growth-associated elements, underscore the complex interplay between genetic traits, metal homeostasis, and physiological performance in genetical modified fish strains.

### 3.2. Correlation Analysis of Trace Elements in Crucian Carp Tissues

This study employed Spearman’s correlation analysis to examine the bioaccumulation patterns of trace elements in muscle and liver tissues in four variants of crucian carp. Statistically significant correlations (*p* < 0.05) were observed in multiple element pairs: WUCI (thirteen positive, four negative), WT (eighteen positive, three negative), FZYJ (ten positive, five negative), and SPYJ (sixteen positive, fifteen negative) (Table 2a–d). Notably, WUCI exhibited exclusively positive correlations among Al, Cr, Ni, Ga, Mo, and Pb, with the strongest positive associations observed for Al, Ni, Ga, As, and Ag, followed by Cr, Rb, Sr, Mo, Pb, and Ba. Silver (Ag) demonstrated the highest number of negative correlations in WUCI, indicating the potential presence of complex toxicity mechanisms.

Strong correlations (|rs| ≥ 0.8) were found to reveal distinct inter-element relationships in certain variants: WUCI showed Ni-Al, Ga-Al/Ni, Sr-As, Ag-Ga/Rb, and Pb-Cr associations; WT displayed Ni-Cr, Ga-Al/Cr, and Ba-Sr linkages; FZYJ exhibited Sr-Rb, Mo-Al/Cr/As, and Pb-Ag connections; and SPYJ demonstrated Cr-Al, Ni-Al/Cr, Ga-Al, As-Cr, Rb-Cr, and Mo-Cr/Ga interactions. These patterns suggest potential metabolic synergism or antagonism among the elements, with variant-specific differences suggesting alterations in physiological metabolism in WUCI [25].

Comparative analysis revealed similar numbers of positive correlations among WUCI (13), WT (18), and FZYJ (10), whereas SPYJ showed markedly more negative correlations (16) than other variants (WUCI: 4, WT: 3, FZYJ: 5). This observation supports the hypothesis of conserved elemental metabolic pathways in WUCI, WT, and FZYJ, whereas SPYJ exhibits divergent mechanisms, as indicted by its unique correlation patterns. Particularly, Ga-As and Sr-Mo pairs showed no significant correlations in WUCI, WT and FZYJ, but exhibited significant negative correlations in SPYJ (*p* < 0.05), further supporting functional conservation among the former three variants [26]. The observed statistical independence of Ba from other elements in SPYJ could suggest its involvement in distinct physiological processes, potentially warranting further investigation. These findings collectively demonstrate variant-specific differences in elemental interactions, which may reflect differential metabolic capabilities between the variants [27].

### 3.3. Assessment of Heavy Metal Contamination Levels

To evaluate heavy metal contamination in the four crucian carp variants, we calculated the pollution index (*P_i_*) for Cr, As, Pb, Cu, and Cd using Equation (1). The results presented in Table 3 showed that *P_i_* values ranging from 0.001 to 0.265 across all samples. Notably, both muscle and liver tissues of WT and FZYJ exhibited clean levels (*P_i_* < 0.2) for all elements analyzed. However, two exceptions were observed: (1) the liver of SPYJ exhibited slightly elevated Pb levels (0.2 < *P_i_* < 0.6), and (2) the liver of WUCI demonstrated marginally higher Cu accumulation (0.2 < *P_i_* < 0.6). In muscle tissue, WUCI exhibited slight contamination for Cr and As, while SPYJ showed elevated Pb levels (0.2 < *P_i_* < 0.6).

These findings are consistent with our previous comparative study of common carp, crucian carp, and grass carp from identical aquaculture environments, where crucian carp showed consistently higher heavy metal accumulation (*P_i_* = 0.163) comparable to the current values of WT (0.189) and FZYJ (0.170) [28]. This pattern may reflect species-specific differences in growth rate, feeding habits, and age-related metabolic processes. Although Cr, As, and Pb are potentially toxic, crucian carp possess detoxification mechanisms to convert these elements into less harmful forms, thereby reducing environmental toxicity at certain concentrations [29].

The variant-specific accumulation patterns suggest distinct metal regulation strategies: SPYJ’s hepatic Pb accumulation likely relates to its enhanced metabolic demands from rapid growth, while WUCI’s muscle Cr/As accumulation may stem from altered metabolic pathways due to bmp6 gene editing. Although all *P_i_* values were below 0.3, certain elements (As, Pb, and Cr) reached mild contamination levels, necessitating further dietary risk assessment.

### 3.4. Potential Health Risk Assessment

The target hazard quotient (*THQ*) and hazard index (*HI*) for As, Cd, Cr, Cu, Fe, Mn, Ni, Pb, and Zn in the liver and muscle tissues of the four crucian carp variants are provided in Table 4. In this study, all *THQ* and *HI* values for both liver and muscle were below 1, indicating an absence of significant non-carcinogenic health risks from individual or combined metal intake through consumption of these freshwater fish. Notably, muscle tissues showed slightly higher *HI* values than liver tissues, primarily due to the presence of Cr and As.

Chromium, an essential cofactor in glucose metabolism, readily binds to muscle proteins, such as insulin receptors and other proteins involved in energy metabolism, participating in the regulation of cellular energy balance. Although the liver serves as the primary detoxification organ for chromium, it is predominantly excreted via bile, leading to reduced residual concentrations in the body [29]. Our previous research demonstrated that the liver of crucian carp efficiently detoxifies arsenic by enzymatically converting As(III) to As(V) or conjugating it with glutathione, facilitating its excretion via bile. In contrast, muscle tissues accumulate higher arsenic levels due to the stable formation of complexes between arsenic and thiol-rich proteins, which impedes the elimination process [30].

Since Cd concentrations were below the detection limit, the calculation of *THQ* and *TCR* calculations was not performed. The highest *THQ* was observed in the WUCI muscle (Cr *THQ* = 0.0648), with HI values ranging from 0.1149 to 0.2432, indicating varying levels of potential health risk. These findings align with prior studies reporting *HI* values < 1 for aquatic products in Northeast China (fish: 0.11–0.226; crustaceans: 0.0905–0.683; amphibians: 0.0658–0.357) [18,28,31].

Target cancer risk (*TCR*) was calculated for As, Ni, and Pb, as these elements are classified as carcinogens. *TCR* evaluates potential lifetime cancer risks associated with carcinogen intake. As shown in Table 4, the calculated *TCR* values for Cr, Ni, As, and Pb were all below 10^−6^, which, according to USEPA guidelines (USEPA, 1987) [20,31], indicates a negligible lifetime cancer, considered acceptable under regulatory standards.

The extremely low *TCR* values (<10^−6^) in this study confirm that the lifetime consumption of these fish poses no significant carcinogenic risk from Cr, Ni, As, or Pb exposure. These results support the overall food safety of the analyzed crucian carp variants, though continued monitoring is recommended to account for potential environmental fluctuations in metal bioavailability, as environmental factors such as water pH, temperature, and pollutants may influence the bioavailability and accumulation of heavy metals in aquatic organisms.

## 4. Conclusions

This study systematically investigates the elemental accumulation and food safety characteristics of four crucian carp varieties: gene-edited intermuscular bone free crucian carp (WUCI) and its sibling wild-type (WT), Songpu silver crucian carp (SPYJ), and Fangzheng silver crucian carp (FZYJ). The results reveal distinct elemental accumulation patterns among the varieties: WUCI shows unique hepatic Mo enrichment and abnormal Rb accumulation, SPYJ displays a specialized elemental regulatory network, while WT and FZYJ exhibit relatively conservative metabolic profiles. Comprehensive health risk assessments (*THQ*/*HI* < 1, *TCR* < 10^−6^) confirm that all varieties meet safety standards, with only minimal contamination observed in specific elements, such as Pb in SPYJ liver and Cr/As in WUCI muscle. Notably, the study identifies a paradox where muscle As levels exceed hepatic concentrations, attributable to the liver’s efficient detoxification mechanisms and the strong binding capacity of muscle tissues. These findings provide scientific evidence supporting the food safety of crucian carp, and establish a theoretical foundation for research on fish elemental metabolism and the breeding of superior varieties with optimized elemental profiles.

## Figures and Tables

**Table 1:** Biometric characteristics of sampled fish by strain.

Strain	Body Weight (g)	Body Length (mm)
WUCI	87.70 ± 19.74	134.69 ± 9.67
WT	70.60 ± 12.43	124.29 ± 6.82
FZYJ	75.51 ± 17.67	128.54 ± 10.50
SPYJ	82.09 ± 18.19	132.31 ± 8.52

**Table 2:** (**a**) Spearman correlation coefficients among trace elements in WUCI strain. (**b**) Spearman correlation coefficients among trace elements in FZYJ strain. (**c**) Spearman correlation coefficients among trace elements in WT strain. (**d**) Spearman correlation coefficients among trace elements in SPYJ strain.

**(a)**											
**Element**	**Al**	**Cr**	**Ni**	**Ga**	**As**	**Rb**	**Sr**	**Mo**	**Ag**	**Ba**	**Pb**
Al	1										
Cr	0.091	1									
Ni	0.952 **	−0.018	1								
Ga	0.830 **	−0.079	0.915 **	1							
As	−0.455	−0.152	−0.358	−0.527	1						
Rb	−0.576	0.006	−0.588	−0.624	0.648 *	1					
Sr	−0.503	−0.176	−0.37	−0.406	0.830 **	0.782 **	1				
Mo	0.006	0.770 **	−0.079	0.018	−0.491	−0.236	−0.455	1			
Ag	0.709 *	0.164	0.721 *	0.806 **	−0.709 *	−0.818 **	−0.709 *	0.479	1		
Ba	−0.564	0.091	−0.43	−0.442	0.745 *	0.479	0.624	−0.248	−0.636 *	1	
Pb	−0.03	0.842 **	−0.091	−0.079	−0.176	−0.236	−0.345	0.673 *	0.188	0.273	1
**(b)**											
**Element**	**Al**	**Cr**	**Ni**	**Ga**	**As**	**Rb**	**Sr**	**Mo**	**Ag**	**Ba**	**Pb**
Al	1										
Cr	0.588	1									
Ni	0.673 *	0.333	1								
Ga	0.200	0.103	0.333	1							
As	−0.782 **	−0.479	−0.515	−0.309	1						
Rb	−0.648 *	−0.358	−0.394	−0.176	0.782 **	1					
Sr	−0.261	−0.297	−0.091	−0.248	0.539	0.818 **	1				
Mo	0.855 **	0.818 **	0.673 *	0.358	−0.806 **	−0.721 *	−0.503	1			
Ag	0.467	0.539	0.527	0.37	−0.745 *	−0.345	−0.164	0.661 *	1		
Ba	−0.479	−0.455	−0.212	−0.224	0.442	0.709 *	0.673 *	−0.624	−0.139	1	
Pb	0.333	0.127	0.624	0.358	−0.552	−0.164	0.152	0.394	0.842 **	0.127	1
**(c)**											
**Element**	**Al**	**Cr**	**Ni**	**Ga**	**As**	**Rb**	**Sr**	**Mo**	**Ag**	**Ba**	**Pb**
Al	1										
Cr	0.697 *	1									
Ni	0.721 *	0.879 **	1								
Ga	0.818 **	0.891 **	0.758 *	1							
As	−0.418	−0.321	−0.127	−0.394	1						
Rb	−0.527	−0.479	−0.394	−0.648 *	0.515	1					
Sr	−0.115	−0.091	0.042	−0.285	0.733 *	0.758 *	1				
Mo	0.673 *	0.661 *	0.564	0.648 *	−0.709 *	−0.697 *	−0.503	1			
Ag	0.624	0.685 *	0.770 **	0.527	−0.006	−0.321	0.212	0.515	1		
Ba	0.345	0.309	0.418	0.176	0.406	0.418	0.806 **	−0.03	0.394	1	
Pb	0.636 *	0.6	0.673 *	0.624	0.345	−0.055	0.503	0.091	0.673 *	0.697 *	1
**(d)**											
**Element**	**Al**	**Cr**	**Ni**	**Ga**	**As**	**Rb**	**Sr**	**Mo**	**Ag**	**Ba**	**Pb**
Al	1										
Cr	0.939 **	1									
Ni	0.818 **	0.939 **	1								
Ga	0.842 **	0.770 **	0.588	1							
As	−0.733 *	−0.830 **	−0.782 **	−0.709 *	1						
Rb	−0.855 **	−0.806 **	−0.770 **	−0.685 *	0.782 **	1					
Sr	−0.733 *	−0.697 *	−0.552	−0.733 *	0.745 *	0.758 *	1				
Mo	0.794 **	0.818 **	0.721 *	0.842 **	−0.867 **	−0.794 **	−0.867 **	1			
Ag	0.588	0.673 *	0.612	0.576	−0.6	−0.527	−0.261	0.588	1		
Ba	−0.042	0.006	0.091	−0.139	0.394	0.212	0.576	−0.321	0.273	1	
Pb	0.721 *	0.673 *	0.624	0.564	−0.37	−0.673 *	−0.394	0.624	0.697 *	0.382	1

* indicates that correlation is statistically significant at 0.05 level (one-tailed), ** indicates significant at 0.01 level (two-tailed).

**Table 3:** Pollution index (*P_i_*) for crucian carp from WUCI, WT, FZYJ, and SPYJ. ^a^ GB2762–2022, ^b^ NY5073–2006.

Elements	Limit Standards	Tissues	WUCI		WT		FZYJ		SPYJ	
			*P_i_*	Pollution Level	*P_i_*	Pollution Level	*P_i_*	Pollution Level	*P_i_*	Pollution Level
Cr ^a^	2 ^a^	Muscle	0.247	Mild	0.189	Clean	0.137	Clean	0.107	Clean
As ^a^	0.5 ^a^	Muscle	0.218	Mild	0.139	Clean	0.170	Clean	0.195	Clean
Pb ^a^	0.5 ^a^	Muscle	0.128	Clean	0.181	Clean	0.122	Clean	0.217	Mild
Cu ^b^	50 ^b^	Muscle	0.014	Clean	0.022	Clean	0.016	Clean	0.047	Clean
Cd ^a^	0.5 ^a^	Muscle	0.001	Clean	0.001	Clean	0.001	Clean	0.001	Clean
Cr ^a^	2 ^a^	Liver	0.159	Clean	0.156	Clean	0.161	Clean	0.150	Clean
As ^a^	0.5 ^a^	Liver	0.038	Clean	0.042	Clean	0.051	Clean	0.036	Clean
Pb ^a^	0.5 ^a^	Liver	0.125	Clean	0.174	Clean	0.117	Clean	0.265	Mild
Cu ^b^	50 ^b^	Liver	0.246	Mild	0.153	Clean	0.108	Clean	0.051	Clean
Cd ^a^	0.5 ^a^	Liver	0.001	Clean	0.001	Clean	0.001	Clean	0.001	Clean

**Table 4:** Risk assessment values (*THQ*, *HI*, and *TCR*) of metal pollutants in tissues of four freshwater crucian carp.

Fish Sample	Target Hazard Quotient (*THQ*)								Hazard Index (*HI*)	Target Carcinogenic Risk (*TCR*)			
	Al	Cr	Ni	As	Sr	Ag	Ba	Pb		Cr	Ni	As	Pb
Liver													
WUCI	0.0373	0.0417	0.0030	0.0249	0.0001	0.0017	0.0001	0.0061	0.1149	1.01 × 10^−6^	1.63 × 10^−6^	1.81 × 10^−7^	3.40 × 10^−9^
WT	0.0430	0.0423	0.0030	0.0331	0.0001	0.0037	0.0001	0.0058	0.1310	1.03 × 10^−6^	1.68 × 10^−6^	2.42 × 10^−7^	3.19 × 10^−9^
FZYJ	0.0412	0.0410	0.0030	0.0234	0.0001	0.0019	0.0001	0.0085	0.1192	9.95 × 10^−7^	1.65 × 10^−6^	1.71 × 10^−7^	4.73 × 10^−9^
SPYJ	0.0452	0.0392	0.0031	0.0237	0.0001	0.0016	0.0001	0.0130	0.1260	9.53 × 10^−7^	1.69 × 10^−6^	1.73 × 10^−7^	7.22 × 10^−9^
Muscle													
WUCI	0.0264	0.0648	0.0019	0.1427	0.0002	0.0007	0.0002	0.0063	0.2432	1.57 × 10^−6^	1.04 × 10^−6^	1.04 × 10^−6^	3.49 × 10^−9^
WT	0.0346	0.0360	0.0027	0.1114	0.0003	0.0010	0.0002	0.0060	0.1922	8.74 × 10^−7^	1.50 × 10^−6^	8.11 × 10^−7^	3.32 × 10^−9^
FZYJ	0.0328	0.0495	0.0028	0.0909	0.0004	0.0029	0.0002	0.0089	0.1882	1.20 × 10^−6^	1.52 × 10^−6^	6.62 × 10^−7^	4.94 × 10^−9^
SPYJ	0.0288	0.0280	0.0023	0.1276	0.0002	0.0008	0.0001	0.0107	0.1986	6.79 × 10^−7^	1.26 × 10^−6^	9.30 × 10^−7^	5.91 × 10^−9^

## Data Availability

The datasets used and analyzed during the current study are available from the corresponding author on reasonable request.

## Supplementary Materials

The following supporting information can be downloaded at: https://www.mdpi.com/article/10.3390/toxics13070595/s1, Table S1: Operation parameters of HPLC-ICP-MS (Agilent 7500 cx); Table S2: Summary of analyte masses, elements for internal standard method (ISTD), analytical conditions for octopole reaction system (ORS), correlation coefficient of standard curve (R), limits of detection (LOD), limit of quantification (LOQ), and results of quality control for study elements; Table S3: Quantifying uncertainty in analytical measurement.
