# Supplementary material for: Accumulation Patterns and Health Risk Assessment of Trace Elements in Intermuscular Bone-Free Crucian Carp
Source: Toxics. 2025 Jul 16;13(7):595. doi: 10.3390/toxics13070595 (PMC12300187; doi:10.3390/toxics13070595)
Supplement: Supplementary file 1 [file toxics-13-00595-s001.zip › toxics-3743608-supplementary.pdf]

Table captions

Table S1. Operation parameters of ICP-MS (Agilent 7500 cx).

Table S2. Summary of analyte masses, elements for internal standard method (ISTD), analytical conditions for octopole reaction system (ORS), correlation coefficient of standard curve(R), limits of detection (LOD), limit of quantification (LOQ) and results of quality control for study elements.

Table S3. Quantifying Uncertainty in Analytical Measurement

Table S1.

Operation parameters of ICP-MS (Agilent 7500 cx).

Operation parameters (optimized daily)	
RF power	1500 W
Reflected power	<15 W
Plasma gas flow rate	15 L min^{-1}
Nebulizer gas flow rate	0.98 L min^{-1}
Auxiliary gas flow rate	0.24 L min^{-1}
Collision gas H_e	4.0 mL min^{-1}
Makeup Gas	0.15 L min^{-1}
Nebulizer Pump	0.10 rps
Uptake Speed	0.40 rps
Uptake Time	45 s
Stabilization Time	30 s
Acquisition	Spectrum(Multi Tune)
Peak Pattern	Full Quant(3)

Table S2.

Summary of analyte masses, elements for internal standard method (ISTD), analytical conditions for octopole reaction system (ORS), correlation coefficient of standard curve(R), limits of detection (LOD) , limit of quantification (LOQ) and results of quality control for study elements.

Analyte	Isotope	ISTD	ORS	R	LOD ($mg\cdot kg^{-1}$)	LOQ ($mg\cdot kg^{-1}$)	RSD (%)	GBW10050 ($mg\cdot kg^{-1}$)	Found value ($mg\cdot kg^{-1}$)	Recovery (%)
Al[a]	27	^{45}Sc	He	0.9998	0.010	0.025	3.32	290	284 ± 9.45	98.1
Cr	53	^{45}Sc	He	0.9999	0.001	0.003	10.5	0.35 ± 0.11	0.38 ± 0.04	109
Ni	60	^{45}Sc	He	0.9999	0.001	0.005	9.61	0.23	0.22 ± 0.021	94.2
Cu	63	^{45}Sc	He	0.9996	0.004	0.010	2.70	10.3 ± 0.7	9.8 ± 0.26	95.2
Ga[b]	71	^{103}Rh	He	1.0000	0.001	0.003	13.4	0.01[a]	0.010 ± 0.001	95.7
As	75	^{103}Rh	He	0.9999	0.001	0.003	7.16	2.5	2.47 ± 0.18	98.8
Mo	95	^{103}Rh	He	0.9995	0.001	0.003	6.56	0.037 ± 0.012	0.034 ± 0.002	98.1
Rb	85	^{103}Rh	He	0.9996	0.001	0.003	6.38	1.4 ± 0.1	1.41 ± 0.09	101
Sr	88	^{103}Rh	He	0.9997	0.001	0.003	4.76	20 ± 2	21 ± 1	105
Ag	107	^{103}Rh	He	1.0000	0.001	0.003	9.37	0.017	0.016 ± 0.002	96.1
Cd	111	^{103}Rh	He	1.0000	0.001	0.003	8.82	0.039 ± 0.002	0.034 ± 0.003	87.2
Ba	137	^{103}Rh	He	0.9996	0.006	0.025	6.51	2.3 ± 0.3	2.4±0.16	104
Pb	208	^{209}Bi	He	0.9986	0.001	0.003	5.56	0.20 ± 0.05	0.18±0.01	90.0

The data are represented as means ± standard deviation.

[a]: The unit of concentration of element is 10^{-2},

[b]: Spiked test sample in 0.010 $mg\cdot kg^{-1}$.

Table S3.

Quantifying Uncertainty in Analytical Measurement

U_a/%	U_b/%					U_c/%	U/%
	U_{b1}	U_{b2}	U_{b3}	U_{b4}	U_{b5}		
3.858	0.07	1	1.4	0.05	1.16	19.20	8.76
0.016	0.07	1	1.4	0.05	1.16	4.31	4.15
0.008	0.07	1	1.4	0.05	1.16	4.31	4.15
0.108	0.07	1	1.4	0.05	1.16	4.32	4.16
0.001	0.07	1	1.4	0.05	1.16	4.31	4.15
0.072	0.07	1	1.4	0.05	1.16	4.32	4.16
0.037	0.07	1	1.4	0.05	1.16	4.31	4.15
0.408	0.07	1	1.4	0.05	1.16	4.48	4.23
0.001	0.07	1	1.4	0.05	1.16	4.31	4.15
0.001	0.07	1	1.4	0.05	1.16	4.31	4.15
0.064	0.07	1	1.4	0.05	1.16	4.32	4.16
0.004	0.07	1	1.4	0.05	1.16	4.31	4.15
0.001	0.07	1	1.0	0.05	1.16	3.35	3.66

Where, U_a: Type A uncertainty; U_b: Type B uncertainty; U_c: Combined standard uncertainty; U: Expanded uncertainty; *k*: Coverage factor, with a value of 2; U_{b1}: Electronic balance; U_{b2}: ICP-MS; U_{b3}: Heavy metal standard solution; U_{b4}: Volumetric flask; U_{b5}: Microwave digestion system.

Calculation formula:

$U_a = SD/\sqrt{n}$,n = 6

$U_b = \sqrt{u_{b1}^2 + u_{b2}^2 + u_{b3}^2 + u_{b4}^2 + u_{b5}^2}$

$U_c = \sqrt{u_a^2 + u_b^2}$

$U = U_c \times k$, $k = 2$
